# P-1991. Unique Demographic Characteristics of Patients Experiencing Long COVID

**DOI:** 10.1093/ofid/ofae631.2149

**Published:** 2025-01-29

**Authors:** Christopher Kricorian

**Affiliations:** For Good Measure, Simi Valley, California

## Abstract

**Background:**

As the COVID-19 pandemic retreats, health care providers now treat many patients experiencing lingering symptoms long after their initial infection, also known as Long COVID (LC). Given that the population of those affected by LC has been generally cumulative, understanding the current demographics of this population is critical to public health planning.

**Methods:**

The US Census Bureau Household Pulse Survey data was used in this analysis. The survey data was collected January 9 - February 5, 2024. Respondents were asked about a variety of demographic factors, as well as their self-reported COVID-19 experiences. The survey was conducted online; N=68,544 respondents were included in the analysis. Demographic factors were compared between those reporting LC, COVID-19 infection that had resolved (Short COVID, or SC), and those who had not experienced COVID (No COVID, or NC). Data were analyzed using X^2^, between group contrast analysis, and non-parametric tests.

**Results:**

Among those surveyed, 16.5% reported experiencing LC, 42.2% reported SC and 41.3% reported NC. Among those who reported LC, 64.3% identified as female (vs. 53.1% SC and 53.0% NC, comparisons to LC p< .05). The median incomes by group were LC $54.6k, SC $75.4K, and NC $59.2K (all pairwise comparisons p< .001). Median age for those with LC was 50 years old, SC was 52 years old, and NC was 60 years old (all pairwise comparisons p< .001). LC individuals were 10.1% Hispanic of any race, compared to SC 8.0% and NC 7.6% (comparisons to LC p< .05). Additionally, 6.3% of those with LC described themselves as Black or African American, vs. 5.7% of SC and 9.6% NC (comparisons to NC p< .05).

**Conclusion:**

Patients experiencing LC have a substantially different demographic profile than both those who experienced SC and NC. LC individuals are younger, poorer, more female, and more likely to be Hispanic. The reasons for these differences are unclear and merit further study, but the general finding that people with LC are more likely to belong to marginalized groups suggests they could have greater difficulty getting adequate care. Reportedly, LC patients may feel their illness is trivialized by others, and these findings underscore the importance for medical personnel to identify and validate LC patient experiences.

**Disclosures:**

All Authors: No reported disclosures

